# The Need for Culturally Responsive Nutritional Counselling for Pregnant Aboriginal and Torres Strait Islander Women in Australia

**DOI:** 10.3390/ijerph22071043

**Published:** 2025-06-30

**Authors:** Lina Jalloub, Stephanie Gilbert, Clare Collins, Marc T. P. Adam, Mieka Thorogood, Tahlia Smith, Janinne Gliddon, Serena St Clair, Nicole Turner, Rhonda Marriott, Roz Walker, Kym M. Rae, on behalf of Deadly Diets Indigenous Steering Committee

**Affiliations:** 1 Indigenous Health Research Group, Mater Research Institute, South Brisbane 4101, Australia; lina.jalloub@mater.uq.edu.au (L.J.); mieka.thorogood@mater.uq.edu.au (M.T.); tahlia.smith@mater.uq.edu.au (T.S.); 2 Faculty of Humanities, Arts and Social Sciences, The University of Queensland, St Lucia 4067, Australia; stephanie.gilbert@uq.edu.au; 3 School of Health Sciences, College of Health, Medicine and Wellbeing, University of Newcastle, Callaghan 2308, Australia; clare.collins@newcastle.edu.au; 4 College of Engineering, Science and Environment, University of Newcastle, Callaghan 2308, Australia; marc.adam@newcastle.edu.au; 5 Ngangk Yira Institute for Change, Murdoch University, Perth 6150, Australia; janinne.gliddon@murdoch.edu.au (J.G.); serena.stclair@murdoch.edu.au (S.S.C.); r.marriott@murdoch.edu.au (R.M.); roz.walker@murdoch.edu.au (R.W.); 6 Indigenous Allied Health Association, Canberra 2600, Australia; 7 Faculty of Health, Medicine, and Behavioural Sciences, The University of Queensland, Herston 4029, Australia

**Keywords:** Indigenous Australians, Aboriginal Australians, Torres Strait Islander Australians, First Nations Australian, perinatal nutrition, dietitians, access to health services

## Abstract

Access to high-quality, culturally responsive nutrition advice during pregnancy is necessary for optimal health outcomes for mothers and babies. Evidence indicates that age, education and access to trained healthcare practitioners have a positive correlation with healthy food intake and positive outcomes. There are limited studies that discuss the importance of providing culturally responsive nutrition advice to pregnant Indigenous women. Therefore, this paper investigates the sources from which Indigenous women access nutrition information, assesses its adequacy in meeting needs, and identifies the effective ways to deliver this information. This study took place in Queensland (QLD), New South Wales (NSW), and Western Australia (WA), which were chosen to represent diverse cultural communities. A total of 103 participants were recruited, including Indigenous women and healthcare practitioners. Focus groups were audio-recorded, transcribed and analysed. Participants indicated that pregnant women are highly interested in improving their nutrition knowledge during pregnancy and actively seek information from their healthcare practitioners and dietitians. Findings suggested dissatisfaction with the information received, as it failed to address their needs. Results of this paper call for an urgent increased presence of community dietitians in antenatal clinics dedicated to Indigenous pregnant women as an additional way to provide families with the information they need for healthy pregnancies.

## 1. Introduction

Pregnant Aboriginal and Torres Strait Islander women (hereafter respectfully Indigenous) and their babies are at a higher risk of gestational diabetes, gestational hypertension, pre-term birth, and low birth weight [1]. Access to high-quality, culturally responsive and necessary nutrition information assists in healthy pregnancy outcomes for Indigenous Mums and Bubs (Bub is an Australian colloquial term for baby). While different nations have different approaches to the delivery of nutritional information during pregnancy, it is not unusual for nutrition information to be delivered by a combination of midwives, dietitians, diabetes educators, general practitioners, or obstetricians. Nutrition during pregnancy can be impacted by food security, religion, financial income, age, and education of the pregnant woman, as well as specific traditions attributed to where they each come from and access to dietitians with expertise in maternal nutrition [2]. Two studies also indicated that older pregnant women, or women with pregnancy intention, are more likely to be encouraged to see their Obstetrician earlier and also tend to have higher nutrition knowledge [2,3].

There is limited information available on what impacts nutritional intake for pregnant Indigenous women. A systematic review conducted globally has suggested that Indigenous women who engage with trained healthcare practitioners, such as their general practitioners (GPs) and midwives and have the provision of timely, effective maternal care have positive health outcomes, including higher birth weights. Both the initiation and duration of breastfeeding occurred at higher rates. However, this study did not disclose if this was exclusive or supplemented breastfeeding [4]. The implementation of the Birthing on Country Services (BOC) for Indigenous Australians utilising a culturally safe, community-led maternity model of care has reduced preterm birth, improved outcomes and reduced cost [5]. Many Indigenous Australians are less likely to attend mainstream health services and engage with trained healthcare practitioners due to geographic isolation and a lack of continuity of care. Australia’s past history of colonisation and ongoing oppression of Indigenous communities has resulted in a lack of trust by communities, ongoing institutional racism, as well as culturally inappropriate and culturally unsafe practices for many Indigenous women during pregnancy care [6].

The lack of culturally responsive care options means that Indigenous women have to fit into healthcare models that were not created for their needs and are at odds with traditional Indigenous ways of pregnancy care, as well as birthing [7]. Women who received antenatal care from primary healthcare services that are delivered by local Indigenous communities, such as Aboriginal Health Services (AMS) and Aboriginal Community-Controlled Health Services (ACCHO), were more likely to rate their care as “very good” compared with mainstream public antenatal care services [8]. This is due to the involvement of a culturally aware workforce, including Aboriginal health practitioners, midwives and general practitioners (GPs). These health services have assisted in providing a safe environment for women to receive health support during pregnancy [8]. It has been highlighted that appropriate healthcare models for Indigenous communities should be embedded in community-based interventions and built upon collaborations with community members; this includes the involvement of Indigenous health practitioners and family members in antenatal care to foster positive health service engagement [4].

However, the published literature available to date does not discuss the role of dietitians in supporting maternity care for pregnant Indigenous women within the Aboriginal Health Service care model. This gap emphasizes the need for a more comprehensive understanding of how nutrition information is delivered and integrated into maternity health services when dietitians provide nutrition support for pregnant Indigenous women. Therefore, this study aimed to investigate the sources from which Indigenous women access culturally responsive nutrition information, assess its adequacy in meeting their needs, applicability in their daily lives, and the most effective way to deliver this information.

## 2. Materials and Methods

### 2.1. Setting

This qualitative study reflects the voices of Indigenous people and was conducted in partnership with several Aboriginal community-controlled health services in three states of Australia: Queensland (QLD), New South Wales (NSW), and Western Australia (WA). Sites were chosen to ensure a widespread representation of Indigenous communities and to showcase the geographical diversity of metropolitan, regional, and rural areas. Additionally, members of the research team had connections to many of the service partners, which greatly facilitated the establishment of this work. In rural and remote QLD and NSW, the locations chosen have AMS and ACCHO, where Indigenous community members receive most of their care. WA focused specifically on community centres in metropolitan areas, Indigenous-specific groups such as playgroups, women’s groups including older women, and Indigenous-community led medical centres. Participants came from the communities of Warwick (Bundjalung lands), Toowoomba (Barunggam lands) and Maryborough (Badtjala lands) in QLD, Moree (Kamilaroi lands) in NSW and Perth (Whadjuk Country) in WA. The research team would like to acknowledge that this study was conducted on Indigenous land, and the practices of the communities are unique to each region of the study. To pay our respect to the traditional owners of the land, traditional Country names will be used to refer to the study sites. We also used capital letters for traditional words, as a way to show respect [9].

### 2.2. Study Design

This paper derived data from a larger study known as the *Mums & Bubs Deadly Diets* research project, which aimed to co-design and co-develop a mobile health (mHealth) nutrition resource for Indigenous women during pregnancy [10]. Indigenous people’s “ways of knowing, being and doing” were embedded into the study methods as per the Australian Institute of Aboriginal and Torres Strait Islander Studies (AIATSIS) Code of Ethics for Aboriginal and Torres Strait Islander Research [11]. As part of the co-design process for the *Mums and Bubs Deadly Diets* study, a large number of yarning groups (similar to a focus group) were undertaken across the sites. “Yarning” is a well-established Indigenous research methodology that is used in qualitative research approaches [12]. When undertaking research, a researcher may set the scene of the yarn at the start of the discussion, and this cultural research approach allows for the sharing of information and stories around a particular topic. However, the range of the discussion is led by the participants rather than the researcher. While the researcher may have a number of prompts to help keep the discussion to a particular topic, the participants will continue the discussion as they see fit. Unlike a semi-structured or structured focus group, a yarn does not have set questions; rather, it is a gentle conversation with shared learnings.

The full study design and its recruitment of participants have been previously described [10]. However, briefly, participant recruitment was conducted in collaboration with each site. Flyers were distributed at each organization, inviting both Indigenous women and healthcare practitioners providing maternity care to Indigenous women to participate [10,13]. In some instances, the research team attended pre-existing groups, such as women’s groups or mums and bubs groups. In some instances, the interviews were conducted rather than yarning sessions, particularly where an individual participant indicated this preference.

Consenting women were invited to attend yarning sessions to share their knowledge of the nutrition support needed within their community, particularly during pregnancy. The focus group utilised social yarning circles to share information and stories with community members. Yarning is a culturally safe and sensitive data collection method that honours Indigenous people’s knowledge by fostering connections and relationships. It facilitates two-way knowledge exchange through storytelling and narrative [14].

### 2.3. Data Analysis

Audio recordings were transcribed by an external service, Digital and Audio Transcription Services (DAATS). Four research assistants (MD, MT, JG, SSC) cross-checked all transcripts against the original audio to ensure accuracy, particularly in capturing all voices and accents correctly. The research team conducted the analysis independently and on hard copy and collaboratively reviewed the results to ensure consistency in the line-by-line coding process and to confirm that no sections were missed in the coding process. This was then translated across to qualitative analysis software (NVivo version 12, 2018), where all transcripts were coded line-by-line.

The senior chief investigator (CI) team consisted of senior researchers that are experts in their field, including as social science (SG), higher education, biomedical, and research knowledge (KMR), nutrition technologies and interventions (CC), Computing and Information Technology (MA), development and validation of new methods for the assessment of dietary intake (MR), Indigenous health and Nursing (RM), and environmental health and infectious diseases research areas and early childhood development (RW).

For the Mums and Bubs Deadly Diets study, data analysis was completed by MT and overseen by SG and KMR to review data collected in QLD and NSW. While in WA, data were analysed by JG and SSC and supported by RW. The data analysis plan was developed primarily by SG and KMR, who ensured that the methods undertaken by both teams were comparable to subject-specific support from the wider investigator team. During the thematic analysis specific to the broader Mums and Bubs Deadly Diets project, it became clear that there were a number of topics that continued to be highlighted by participants as a priority, and this paper is the result. LJ and MT, alongside SG and KMR, reviewed data from all sites with this in mind. LJ (both a research assistant and clinical dietitian) collated and led the writing of this paper.

### 2.4. Ethics Approval

The project was approved by Mater Misericordiae (HREC/MML/62512 Approved 23 July 2021), University of Queensland (2021/HE002031 Approved 6 September 2021), Aboriginal Health and Medical Research Ethics Committee of New South Wales (1966/22 Approved 12 August 2022), and Western Australia Aboriginal Health Ethics Committee (HREC1102 Approved 29 November 2021). The *Mums and Bubs Deadly Diets* Indigenous Steering Committee (ISC) was set up as part of this study to work closely with and oversee this project in data collection, analysis, and writing to ensure that data analysis and reports are accurate and representative of the community.

### 2.5. Aim

The aims of this specific study were to understand pregnant Indigenous women’s views about access to dietitians and adequacy in meeting their nutritional needs. In order to address these aims, a sub-analysis of the main Mums and Bubs Deadly Diet qualitative data was undertaken.

## 3. Results

A total of 103 participants took part in 16 focus group sessions and six interviews; 64 were community members, and 39 were healthcare practitioners (HCPs) who provide care to pregnant Indigenous women (with some identifying as Indigenous community members themselves). Healthcare practitioners encompassed midwives, Indigenous health practitioners, Aboriginal health workers, dietitians, health services staff, and nurses.

**Figure 1:**
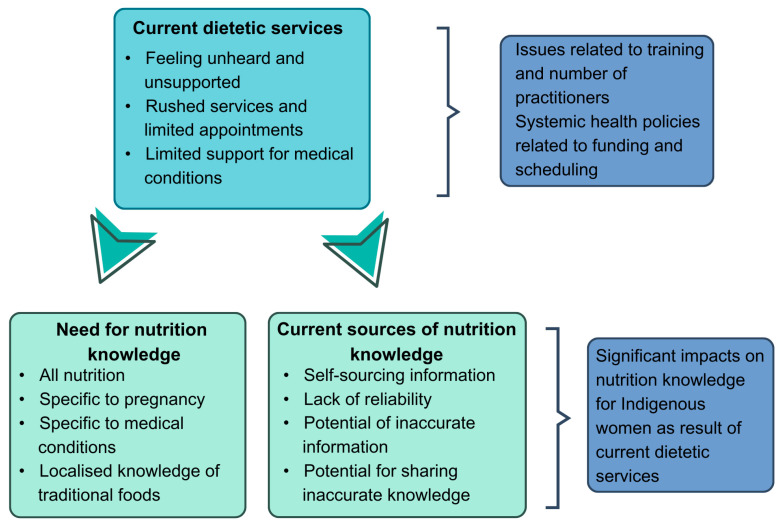
The three themes for this study are related to systemic health policies that impact on current dietetic services. This consequently has downstream impacts on the community’s need for nutrition knowledge and their current sources of information.

When undertaking the sub-analysis of the dataset to understand perceptions of culturally responsive nutritional advice during pregnancy, three main themes were identified. These three themes were (1) improving nutrition knowledge, (2) dietitian services, and (3) sources of information. Figure 1 highlights how these three themes are connected. Quotes included within this study come from across all listed regions; however, they are not attributed to any specific location, as the size of some communities increases the potential for identification for either the participant or health practitioners within a community.

### 3.1. Improving Nutrition Knowledge

In the yarning sessions, women expressed their motivation to improve their knowledge of nutrition to protect their babies and have positive pregnancy outcomes. They wanted to improve their knowledge on how to control cravings, what foods to eat, recipes, money-saving tips, and how to manage their health conditions using food.

*“[More information] on healthier eating. Cause, man, I could eat a whole thing of ice cream.”*

—Community member

They have also shared with us that during pregnancy, they try to eat healthily and improve their nutritional intake, and some of these habits stay with them post-pregnancy.

*“Once I did find out I was pregnant, I did put a lot more greens in my diet and fruit.”*

—Community member

Non-Indigenous healthcare practitioners shared that women are interested in learning more to improve their own health and that of their children. They acknowledged that women’s pregnancy journey is impacted by intergenerational trauma due to forced relocation, land dispossession, and loss of spiritual practices, language, and culture.

*“And they’re learning—trying to learn how to be a good mum or how to be pregnant with no help or guidance like, you know. Now this generation’s actually got guidance where the last generation had no guidance about how to be pregnant. So instead of having like, here are some diseases and problems, it’s more like how to have a better pregnancy than your mum had.”*

—Non-Indigenous HCP

An Indigenous woman from Maryborough (Badtjala lands) expressed a desire for hands-on, practical nutritional information. She told us that she aimed to enhance her skills and needed information to better provide for herself and her growing family.

*“[wants information on]—what’s your normal plate? That? You know, and—and the healthy—the healthy size is a piece of meat the size of your bread.”*

—Community member

### 3.2. Sources of Nutrition Information

Many of the Indigenous mothers who participated expressed a lack of information about nutrition during pregnancy. They also advised us that there was limited support when they sought out nutrition information. As a result, alternative information solutions were self-sourced from mobile apps (e.g., MyFitnessPal, Calorie King), support groups on social media platforms (e.g., Facebook groups), and in-person community mothers’ groups.

*“I’ve got quite a few pregnancy and mum support groups on Facebook. And, you know, you’ve got so many women answering you, and giving you different kind of support.”*

—Community member

Some of the information they have been sharing through these mechanisms is about using traditional foods that are local to their community. In these regions, Indigenous women have been supporting each other, learning from each other’s experiences, and sharing the information they have.

*“Kangaroo. You can’t get a better source of iron than… kangaroo.”*

—Community member

Similarly, in Perth (Wadjuk Country), a metropolitan region, Indigenous women have shared that their main sources of information are family support and social media. However, they struggled to access ACCHOs within their area as they were not aware of them.

*“I went through an [ACCHO], and I didn’t know about them until a family member told me.”*

—Community member

However, the inability to source evidence-based nutrition information can, at times, result in the circulation of unsafe and incorrect information and incorrect identification of safe foods during pregnancy, e.g., avoiding nutritious foods and incorrect serving sizes.

*“I avoided, oh, a lot of citrus fruits. ‘Cause apparently they causes miscarriage, and I’ve had three stillborns.”*

—Community member

### 3.3. Dietitian Services

Seeking evidence-based nutrition information from a local dietitian can be challenging for many reasons. Often, the information pregnant Indigenous women have been receiving is not tailored to their individual needs. A participant from Warwick (Bundjalung lands) shared that despite seeing the dietitian, she felt ill-equipped to make healthy lifestyle choices because the information shared was not practical.

*“I think another thing that’s needed is … more information about gestational diabetes. ‘Cause the dietitian gives you just a basic amount, and you sit there racking your brain, ……I got diagnosed yesterday, day before, and I went to the shops and I was looking at the nutrition information, and I was like I don’t know what I’m supposed to eat, what I’m not supposed to eat. And it’s really hard to make good meals when, you know, no carbohydrate, no sugar.”*

—Community member

Across all regions, women reported experiences of feeling judged or labelled by healthcare practitioners. This included experiences when birthing, visits to hospitals, general practitioners, and dietitians.

*“like I had knee problems, and, [the HCP] “oh, it’s ‘cause you’re fat”. Just so rude, hey. Just so mean.”*

—Community member

Additionally, women shared stories of feeling unheard when visiting the dietitian within the hospital setting and were rushed out of the appointment quickly.

*“the hospital dietitian is like they want to get you in, out. They don’t really care about your health.”*

—Community member

There were instances where referrals to a dietitian were not made, and health practitioners relied more on prescriptive medications. An Indigenous woman told the story of how she wanted to manage vitamin C deficiency using nutrition; however, the practitioner made the decision to prescribe a supplement without consulting her or understanding her needs.

*“Well, I craved a lot of, um, like orange. Orange things. And then the doctor looked up the thing and goes you’re low in, um, vitamin C. And he goes, get on vitamin C tablets and you’ll be fine. And I was like, yeah, but I like eating the fruit.”*

—Community member

Non-Indigenous healthcare practitioners shared that pregnant women, including Indigenous women, generally do not have enough time with the dietitian. A midwife participant shared that in the community she works in, pregnant Indigenous women generally access dietitians through the hospital system only, as these are without cost and could only do so if they have chronic health conditions. She explained that women with gestational diabetes (GDM) see the dietitian for one visit only at the hospital with no follow-up appointment. The main source of contact is a diabetes educator, who has a scope of practice to monitor insulin and medication intake with limited capacity/time for education. Many midwives have had to cover this knowledge gap by providing nutrition information to support women during their pregnancy.

*“Um, but with us, they actually have—when they get diagnosed with GDM or if they’ve got, you know, um, Type 1 diabetes, they actually see the diabetes educator at the hospital in antenatal clinic and they have a diabetes, um—they have the—the dietitian, um, do a bit of a talk to them and sort of give them brochures on the food. We don’t actually sort of—they get that information directly from the hospital, but we sort of support them after that in a way of, you know, …—probably shouldn’t have had that food, you know, because yes, that will spike it and you know, maybe try whole meal or bread before you go to bed or whatever, you know. …, but yeah. So it—it’s kind of the hospital that education initially.”*

—Non-Indigenous HCP

A clinician explained that they have been providing nutrition education information using a brochure to pregnant Indigenous women. Other clinicians who participated in the project supported this and informed us that they provided information using brochures, flyers, videos, and text message reminders.

*“We give them a brochure…, it’s like red food, um, fruit food, orange food, that sort of thing. And it does say food to avoid in pregnancy and it’s highlighted in red.”*

—Non-Indigenous HCP

## 4. Discussion

This study aimed to explore the sources that pregnant Indigenous women use to access culturally responsive nutrition information, assess its adequacy in meeting their needs, applicability in their daily lives, and the most effective way to deliver this information. Findings suggest that pregnant Indigenous women are very interested in improving their nutrition; they have been accessing several avenues to obtain this information, such as social media, community and relying on healthcare practitioners. However, many of the women shared that they do not feel supported by the dietitian they see in the community; they feel rushed at the appointment, unheard and judged. This has resulted in misinformation and untailored nutrition advice circulating.

During the yarning sessions, Indigenous women advised that the information they receive is not culturally responsive or designed to foster growing their own knowledge and capacity. They advised that clinicians, specifically dietitians, are using deficit, negative and culturally unsafe language by shifting blame to pregnant Indigenous women without understanding their perspective, views, and medical history. Evidence shows that delivering culturally responsive care and building rapport requires time [15]. However, many dietitians, including hospital dietitians, are faced with a heavy workload and time constraints, which limit their capacity to build relationships and deliver culturally responsive care. Unfortunately, this can be perceived by patients as a lack of care and support rather than the reflection of systemic issues, making it unlikely that patients will willingly seek out help from the same clinician.

Healthcare practitioners such as midwives, nurses, and dietitians have reported that they feel poorly equipped to provide culturally responsive maternity care to Indigenous Australians due to insufficient education and training [7]. It has been suggested that many health practitioners are limited by their own cultural experience, beliefs, and practices, with little understanding of the institutional racism and discrimination inside the health service or within society in general [16]. This may be due to the insufficient education within the university curriculum for midwives, nurses, and allied health (specifically dietitians), which does not challenge them to understand the worldviews of cultures other than their own [17]. A study conducted with new graduate dietitians in Australia also identified that dietetic students are not getting adequate cultural training in universities. The study suggested that this is due to Indigenous cultural training being delivered by non-Indigenous lecturers and often as part of larger lectures on cultural competence across a broad range of cultures [18]. This study highlighted that new graduate dietitians who have never had the opportunity to do a placement in Indigenous communities lack skills at building rapport, and lack the understanding of Indigenous health disparities. Australia has made limited progress in providing culturally safe care for pregnant Indigenous women, and there are limited guidelines or policies that encompass cultural safety and care provision in maternity services [17].

Dietitians Australia reported that in 2020, there were 7428 dietitians, with only 32 (0.4%) who self-identified as Aboriginal and/or Torres Strait Islander [19,20]. This indicates that the majority of dietitians in Australia are non-Indigenous, and less than 1% of the workforce identifies as Indigenous [21]. The lack of Indigenous-trained dietitians highlights that the Indigenous pregnant women’s main sources of contact with dietitians are non-Indigenous dietitians. A national strategy is likely to be required within Australian academic institutions to recruit Indigenous students into dietetics training to overcome this gross disparity within the workforce. All dietitians are trained within an evidence-based paradigm that prioritises Western knowledge systems and biomedical sciences, which are at odds with Indigenous people’s traditional birthing practices and knowledge sharing [17,22]. This suggests that dietitians are not adequately prepared to work with Indigenous communities, nor across worldviews and knowledge systems that differ from how they were trained to approach their practice [23]. This has been supported by a study conducted with non-Indigenous dietitians; the participants reported that cross-cultural consultation is “too hard” and dietitians were “too scared” to try working across cultural divides [23]. This disparity arises from the varying approaches to Indigenous health education among universities, as they have different curricula in the way they provide cultural education, as there is no consensus on how to do it right and challenge the students’ worldviews [21]. Additionally, there is a lack of evidence around effective workforce development strategies being utilised to upskill dietitians to work collaboratively with the Indigenous communities [23]. The participants of our study advised the study team that they were highly interested in improving their health and nutrition; however, the dietitians they interacted with did not provide them with the information they needed.

The healthcare practitioners who participated in the study agreed that this is due to the current healthcare models available in many health services that provide care to pregnant Indigenous women. They advised that pregnant women are only able to see a dietitian upon a confirmed diagnosis of a medical condition, rather than before a health complication arises. The hospital dietitians and community midwives have attempted to cover and address this gap using the available resources that each of their professions can access. However, globally, dietitians are understaffed and overworked in hospitals, where the workforce is only about half of what is required, and they are struggling to meet the public demand [23]. Meanwhile, midwives do not have the scope of practice to provide tailored nutrition. 

The current literature indicates that pregnant Indigenous women are not meeting the Australian Guide for Healthy Eating (AGHE) daily food group serving recommendations for pregnant women [24]. The study suggested that the nutrient intakes of many pregnant Indigenous women are also inadequate compared with pregnancy requirements. However, the women in this study highlighted that pregnancy increased health-seeking behaviour, and women are more interested in improving their knowledge of nutrition. Despite the limited research in this space for pregnant Indigenous women, a systematic review conducted globally supported the findings of this study, including women’s eating behaviour changes, showing a significant increase in fruit and vegetable consumption, a decrease in egg consumption, a decrease in fried and fast food consumption and a decrease in coffee and tea consumption [3].

In our study, women advised that their main source of nutrition information is their midwives due to the limited access to dietitians in their communities. This issue was particularly apparent in the sites of rural and remote Indigenous communities. This finding has been supported by an Australian study, which suggested that midwives are the main source of health information for pregnant women [25]. Providing nutrition education during pregnancy is within the midwife’s scope of practice as per the International Confederation of Midwives Essential Competencies [26]. Their scope of practice is to assess nutritional status, provide counselling about nutrient supplement intake and dietary intake, and assess women’s nutritional intake [26]. However, midwives are not trained to provide tailored nutrition advice in chronic condition management for various health conditions or to support women to make behavioural changes.

Additionally, a literature review conducted in 2013 suggests that Australian midwives lacked basic knowledge of the nutrition requirements for pregnant women [27]. This has been attributed to the inadequate nutrition education provided in undergraduate and postgraduate midwifery programmes. This finding was further explored in a survey conducted through the Australian College of Midwives [25]. Most of the nutrition education received occurred through professional development opportunities initiated by midwives, which indicates that they are keen on improving their nutrition knowledge.

Across Australia, there are limited public health nutrition interventions that specifically include the involvement of dietitians [28]. Therefore, although women are interested in learning more, they have limited access to public health nutrition education programs during pregnancy. Moreover, research shows that public health nutrition interventions generally do not focus on pregnancy, and they have also suggested that there is a limited program that focuses specifically on pregnant Indigenous women [29]. Furthermore, many of the available interventions within Australia do not involve dietitians. This finding was further explored, suggesting that public health interventions that included dietitians had better health outcomes and more uptake by the public [28]. This indicates that pregnant women are not receiving adequate nutrition education to make informed decisions during their pregnancy. Therefore, the literature suggested that the most effective way of increasing knowledge during pregnancy is through individualised dietitian sessions [28].

Pregnant Indigenous women who participated in this study and their healthcare practitioners advised that there is limited access to dietitians in the community, and their main point of contact is through hospitals. The findings of this study align with previous studies that suggested an inequitable distribution of the dietetic workforce and that dietitians (both private and public) are concentrated in affluent metropolitan centres and high-income postcodes. This then leads to limited access to dietitian intervention in low-income postcodes [23]. The National Aboriginal and Torres Strait Islander Health Plan (2013–2023) highlighted a need for improved quality and accessibility of routine antenatal care, as well as provision of evidence-based strategies to reduce maternal stress and smoking, improve nutrition in pregnancy, and increase breastfeeding [30]. Likewise, the National Strategic Directions for Australian Maternity Services report also reinforces the need for culturally safe and accessible perinatal care for Indigenous women [31]. However, the findings of this current study highlight that there is still a significant way to go to create the changes needed to support Indigenous women’s needs during pregnancy.

The current literature suggests that Australians are unable to afford fees to consult private health practitioners, including dietitians [23]. To access dietitians through the public health system in Australia, they would need to use the Medicare Benefits Scheme (MBS), the National Disability Insurance Scheme (NDIS), and/or hospital services. Private health insurance is another avenue for community members to access a dietitian. However, it does not cover dietitian consultation in the basic cover, and some private health insurance companies may require paying more to include this coverage. MBS and NDIS enable community members to access health services subsidised by the Australian Government within their community [32,33]. MBS assists community members in accessing a dietitian if they have a chronic condition, mental health and/or eating disorder, while NDIS helps people with disability. Community members are typically entitled to 5–10 visits per year, with each session lasting about ~20 minutes. These services are often shared with other allied health professionals, meaning that only 2–3 dietitian sessions may be allocated annually. The allocation of referrals to hospital dietitians and community dietitians is usually determined by the general practitioner or the NDIS case planner and is highly dependent on whether the decision-maker deems it appropriate and the complexity of the medical condition [34]. However, in many cases, those referrals are never made, and many patients are unaware of them or have to argue their cases and their need for the decision-makers to be able to access them [35]. The number of dietitians who accept Medicare referrals without co-payment is limited because they are not financially viable, as the MBS does not provide sustainable income, and the NDIS eligibility criteria are challenging. The hospital system is unable to meet the high demand, and patients are sometimes put on long waiting lists before they can see a dietitian [35]. Furthermore, none of the services listed covers pregnancy-related nutrition support, leaving pregnant women in those Indigenous communities with limited or no access to publicly funded dietitians in their communities.

### Strengths and Limitations

This study has several strengths and limitations. Strengths included the utilization of Indigenous-specific methodology that engaged Indigenous women of reproductive age and their healthcare practitioners [13]. Amongst these methodologies was the use of yarning-centred methods, which encouraged the participants to be part of a safe environment to share their stories and diverse experiences in accessing dietitians and nutrition resources [36]. It also provided a unique perspective across diverse Indigenous communities of Australia in metropolitan, remote and rural locations.

This study ran during COVID-19, which restricted the team’s movement and access to communities and extended the project timeline more than anticipated. This limited the number of practitioners, and the restriction of movement also severely impacted communities themselves. Further, it is worth noting that while participants voluntarily consent to participate, there is likely a self-selection bias that must be acknowledged. As with much research, the research team was left with more questions to investigate. For instance, exploring the lack of access to medical practitioners or dietitians, particularly with knowledge about Indigenous peoples’ lives, was a finding that warrants further investigation.

Additionally, participants shared that they are highly interested in improving their nutrition, and they highlighted some major challenges in accessing dietitians, but these challenges could withstand further investigation from the women’s perspective. In this study, this meant a reliance on the existing literature and research to fill this gap.

## 5. Conclusions

Pregnant Indigenous women require evidence-based nutrition education to support women during the antenatal and postpartum stages of pregnancy. However, this study has identified that the best opportunities offered have mostly been self-sourced online research and community groups. There have been limited culturally responsive dietitian interventions to support pregnant Indigenous women. This paper advocates that policy-makers consider the development of a publicly funded maternity care model that includes dietitians who will assist in the creation of routine culturally safe nutrition care for Indigenous women during their pregnancies. This will provide practical actions to support the advocacy as planned by the National Aboriginal and Torres Strait Islander Health Plan and the National Strategic Directions for Australian Maternity Services [30,31] This will likely lead to an increase in dietitian-led public health interventions to support strong pregnancy outcomes. To provide this support, dietitians require extensive cultural training as a part of their university curriculum, and we suggest that this becomes widely implemented within the health education sector. Future research areas, such as those identified to speak to pregnant women themselves, should lead to the creation of evidence-based dietitian practices and their applicability in Indigenous communities.

## Data Availability

Approval from the Deadly Diets Indigenous Steering Committee and the appropriate Ethics committee would be required in order to access data from this study.

## Abbreviations

The following abbreviations are used in this manuscript:

GP	General Practitioner
BOC	Birthing On Country
AMS	Aboriginal Medical Service
ACCHO	Aboriginal Community-Controlled Health Organisation
QLD	Queensland
NSW	New South Wales
WA	Western Australia
AIATSIS	Australian Institute of Aboriginal and Torres Strait Islander Studies
HCP	Healthcare professional
GDM	Gestational Diabetes Mellitus
AGHE	Australian Guide to Healthy Eating
MBS	Medicare Benefits Scheme
NDIS	National Disability Insurance Scheme
